# The Role of Breast Cancer Stem Cells in Chemoresistance and Metastasis in Triple-Negative Breast Cancer

**DOI:** 10.3390/cancers13246209

**Published:** 2021-12-09

**Authors:** Lin He, Neda Wick, Sharon Koorse Germans, Yan Peng

**Affiliations:** 1 Department of Pathology, University of Texas Southwestern Medical Center, 6201 Harry Hines Blvd, Dallas, TX 75235, USA; lin.he@phhs.org (L.H.); NEDA.WICK@phhs.org (N.W.); SHARON.GERMANS@phhs.org (S.K.G.); 2 Harold C. Simmons Comprehensive Cancer Center, University of Texas Southwestern Medical Center, 5323 Harry Hines Blvd, Dallas, TX 75235, USA

**Keywords:** cancer stem cells, breast cancer stem cells, tumor biomarker, triple negative breast cancer, chemoresistance, metastasis

## Abstract

**Simple Summary:**

Triple negative breast cancer lacks targeted therapies and has poor prognosis; chemotherapy is currently the main treatment modality. Growing evidence has shown that breast cancer stem cells are associated with tumor initiation and metastasis and may play a critical role in chemoresistance. Multiple targets against breast cancer stem cells are now under investigation. Recent advances in the role of breast cancer stem cells in triple negative breast cancer and the identification of cancer stem cell biomarkers have paved the way for the development of new targeted therapies. The discovery of potential molecular signaling pathways targeting breast cancer stem cells to overcome chemoresistance and prevent metastasis will improve the overall survival of patients with triple negative breast cancer.

**Abstract:**

Triple negative breast cancer (TNBC) remains an aggressive disease due to the lack of targeted therapies and low rate of response to chemotherapy that is currently the main treatment modality for TNBC. Breast cancer stem cells (BCSCs) are a small subpopulation of breast tumors and recognized as drivers of tumorigenesis. TNBC tumors are characterized as being enriched for BCSCs. Studies have demonstrated the role of BCSCs as the source of metastatic disease and chemoresistance in TNBC. Multiple targets against BCSCs are now under investigation, with the considerations of either selectively targeting BCSCs or co-targeting BCSCs and non-BCSCs (majority of tumor cells). This review article provides a comprehensive overview of recent advances in the role of BCSCs in TNBC and the identification of cancer stem cell biomarkers, paving the way for the development of new targeted therapies. The review also highlights the resultant discovery of cancer stem cell targets in TNBC and the ongoing clinical trials treating chemoresistant breast cancer. We aim to provide insights into better understanding the mutational landscape of BCSCs and exploring potential molecular signaling pathways targeting BCSCs to overcome chemoresistance and prevent metastasis in TNBC, ultimately to improve the overall survival of patients with this devastating disease.

## 1. Introduction

Stem cells are characterized by their self-renewal capacity and potential to differentiate into many more specialized mature cell types. A subpopulation of stem-like cells within tumors, known as cancer stem cells (CSCs), have been identified to exhibit characteristics of both stem cells and cancer cells. CSCs in human breast tumors were initially identified in 2003 by Al-Hajj et al. [1]. Since then, a number of cell surface markers such as the clusters of differentiation (CD) markers CD44 and CD24 and aldehyde dehydrogenase 1 (ALDH1) have been utilized to isolate/enrich CSCs in order to further characterize their behavior [2]. Marker panels have been studied in various tissue subtypes to isolate organ-specific tumor stem cells. The CD44+/CD24−/ALDH+ phenotype in breast cancer cells has been recognized with an increased potential of tumorigenicity [3]. Other CSC markers, including ATP Binding Cassette Subfamily G Member 2 (ABCG2) and CD133, have been investigated [4,5,6]. Breast CSCs (BCSCs) play a major role in breast cancer invasion and metastasis [7], with the tumor microenvironment being a critical factor for growth signaling and proliferation [8,9]. In addition to breast cancer, CSCs have been associated with the prognosis of other tumors such as medulloblastoma, lung cancer and prostate cancer [10].

Breast cancer consists of four major molecular subtypes: luminal A (Hormone receptor (HR)+/HER2), luminal B (HR+/HER2+), HER2-enriched (HR−/HER2+) and triple-negative or basal-like (HR−/HER2−) [11,12,13]. Triple negative breast cancer (TNBC) accounts for about 15–20% of all breast cancers. TNBC often has basal-like morphology, has an epidemiological predilection for younger female and African American or Hispanic ethnicity [12]. Patients with TNBC usually have poor prognosis compared to ER+ and/or HER2+ breast cancers. However, like other breast cancer subtypes, chemotherapy is still the primary treatment option for TNBC. Studies have shown that patients undergoing neoadjuvant chemotherapy have significantly worse survival rates in TNBC subtypes as compared to non-TNBC subtypes, particularly in the first 3 years after initial diagnosis [14]. TNBC also has been found to be associated with an increased risk for early metastasis, compared to other types [15]. There is a pressing need to study the tumor biology of TNBC, the mechanisms of metastases, and therapy resistance. More so, there is a need for effective targeted therapies against chemotherapy-resistant TNBC.

It has been suggested that CSCs play an important role in the tumorigenesis and tumor biology of TNBC. Like other organs and tissues, CSCs originate from normal stem cells in breast tissue with increasing capacity for self-renewal and proliferation. CSCs, if present in TNBC, can enhance a tumor’s capacity for metastasis and risk of invasion [8]. Studies have shown that CD44+/CD24− and ALDH1+ breast cancer stem cells are enriched in TNBC and may contribute to the propensity of TNBC for chemoresistance and tumor metastasis [16,17]. This tumor evasion mechanism from chemotherapy is likely to play a more important role in tumorigenesis and outcome in TNBC compared with non-TNBCs such as luminal A type breast cancer. CSCs can potentially serve as key regulators, driving the aggressiveness of TNBC [18].

Although uncertainties about BCSCs exist, it has been suggested that CSCs should be considered as possible therapeutic targets. Emerging evidence demonstrates that CSCs usually escape current anti-cancer therapies, which may partly contribute to metastasis dissemination, tumor recurrence and poor prognosis [18,19]. Eliminating breast cancer stem cells may potentially improve the prognosis of TNBC. Recent research has focused on the immunohistochemical and genomic signatures for potential biomarkers and targets specific to BCSCs. These potential markers could be the crucial information needed to guide treatment choices and predict sensitivity and response to therapy. Several clinical trials have aimed to target breast cancer stem cells with different proposed approaches. Specific drugs to Notch, Sonic hedgehog (Shh) and Wnt signaling pathways of BCSCs are under investigation [20]. Immunotherapies, such as monoclonal antibody against CD44, and gene-edit technology such as Clustered Regularly Interspaced Short Palindromic Repeats (CRISPR) are other potential treatment options that specifically target BCSCs [21].

## 2. Cancer Stem Cells in Breast Cancer

BCSCs are a small cell subpopulation among all tumor cells, with unique stem cell characteristics such as self-renewal, high proliferation and differentiation potential to multiple lineages within the breast. BCSCs interact with their tumor microenvironment (TME) as well as on the inducing factors and elements. Markers that have been identified in BCSCs include ALDH1+, CD24− and CD44+. Cells which simultaneously express these markers have the highest tumor initiating and proliferative capacity. Several theories have been proposed about the origin of BCSCs. One of them suggests mutations in dormant normal stem cells results in their transformation to cancer stem cells, a process similar to that of non-stem cells [22,23].

Several markers have been used for the isolation and identification of BCSCs. In 2003, Al-Hajj M et al. first described and isolated the BCSC phenotypes CD24− and CD44+ [1]. CD24 is a cell surface glycoprotein, which has been shown to play a role in tumor progression and metastasis. As a ligand for P-selectin, CD24 is proposed to interact with endothelial cells and platelets during metastasis and ultimately has been linked to poor prognosis and decreased survival [24,25]. As another cell surface glycoprotein and specific receptor to hyaluronan, CD44 plays a key role in breast cancer adhesion, motion, migration and invasion, all of which have a significant impact on early tumor metastasis. [26] ALDH1 is a recently described BCSC marker which belongs to a family of cytosolic enzymes involved in oxidation of intracellular aldehydes that convert retinaldehydes to retinoic acid [27].

Six molecular subtypes of TNBC have been proposed: immunomodulatory, mesenchymal, mesenchymal stem-like, luminal androgen receptor and two basal-like subtypes [28]. The CD44+/CD24− lineage cells generally possess a mesenchymal or myoepithelial-like phenotype and are found more peripherally at the tumor edge. ALDH1-expressing BCSCs have a more epithelial or luminal phenotype and are located more centrally in tumors. These characteristics enable effective epithelial-mesenchymal transition and vice versa [29]. This explains the increased rate of metastasis in BCSC enriched tumors.

Cancer cells with the CD44+/CD24−/ALDH1+ phenotype in breast cancer can therefore be distinguished from other cancer cells by their stem-like features, ability to maintain survival and the role in cancer invasion and metastasis, particularly in TNBC.

## 3. Breast Cancer Stem Cell Regulation Pathways in Triple Negative Breast Cancer

TNBC tumors are known as being enriched for BCSCs. Transcription factors, signaling pathways and tumor suppressor genes have played a pivotal role to maintain the state of stemness of BCSCs. The Wnt, Notch, and Shh pathways have been identified as playing an important role in BCSC and TNBC biology [20,30,31]. Like in many other epithelia, Wnt pathway activity is critical for stem cell self-renewal and multipotency in the breast [32]. Although canonical Wnt signaling is the most well-known path to β-catenin stabilization, numerous other signaling pathways also regulate β-catenin stability and transcriptional activity [33]. For instance, the β-catenin mediated Wnt pathway has several downstream targets including MMP7 and PTEN, as characterized by Dey and colleagues [34]. In TNBC, differential activation of the Wnt pathway correlates with increased MMP7 and decreased expression of PTEN. Patients with increases in MMP7 have a lower rate of pathologic complete response and a high residual disease burden [35]. Inhibition of the Wnt pathway leads to decreased proliferation and induction of apoptosis, bringing to light possible therapeutic targets [36]. Additionally, wnt10B/β-catenin modulates HMGA2, and expression of HMGA2 has been shown to predict the metastasis rate and relapse-free survival [37]. Sulaiman and colleagues found that increased Wnt and histone deacetylase (HDAC) activities are associated with loss of ER and PR expression, poor survival, and increased relapse in patients with invasive breast cancer. Furthermore, in a subset of TNBC cell lines, Wnt signaling and the repression of ER and PR was found to be inversely correlated [38].

The canonical Notch signaling pathway is initiated by activating Notch receptors upon binding to Serrate- and Delta-like ligands present on the cell membranes of adjacent cells. Notch receptors have intracellular domains that are releases into the nucleus after proteolytic cleavages. These Notch intracellular domains can recruit MAML1 and histone acetyltransferase p300 to form active transcriptional complexes, which is the final regulator of the Notch target genes [39]. It was found that the Notch 4, one of the four Notch receptors, is involved in the constitutive ligand-independent activation of Notch 4, and could facilitate the development of mammary adenocarcinoma [40]. BCSCs ectopically expressing Notch 4 in TNBC have shown increased proliferation and invasiveness, whereas inhibition/knockdown of Notch4 decreases cell proliferation, invasion, tumor volume, and tumorigenicity [39].

The canonical Shh pathway is activated by releasing its ligands to bind and inhibit transmembrane receptor Patched-1 (PTCH1). PTCH1 can subsequent initiate an intracellular signal cascade to activate of the downstream transcription factors of glioma-associated oncogene (GLI) [41]. Common Shh target genes include *Cyclin D1/2* [42], *PDGFR*, *MYC* [43], *BCL2* [44], *VEGF* [45], *MMP9* [46] and *SOX2* [47]. The non-canonical Shh pathway, on the other hand, does not reply on the PTCH1 to activate GLI. Instead, it can cross talk with a variety of other signaling cascades [41]. Of note, Shh pathway activation is involved in the TME of the CD44+/CD24−/ALDH1+-expressing BCSCs. Shh pathway activation has been found to mediate the self-renewal of BCSCs after radiation or chemotherapy, which, not surprisingly, results in therapy resistance.

**Figure 1:**
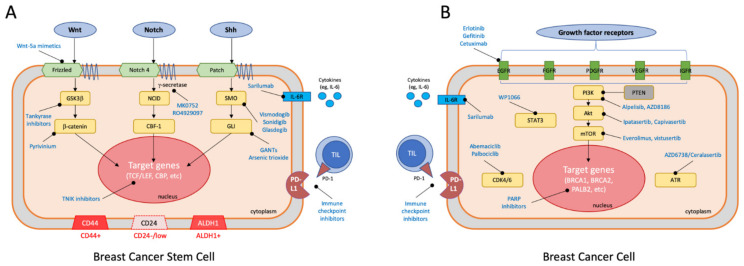
Summary of CSC specific mechanisms and pathways targeting breast cancer stem cells (**A**) and non-CSC specific mechanisms and pathways targeting breast cancer cells (**B**).

The self-renewal mechanism of CSCs, involving the signal transduction pathways noted above and including Wnt, Notch, and Shh [48], is illustrated in Figure 1A. Further investigation of these signaling pathway functions and their interactions with other pathways can aid the development of CSC-targeted therapy for the treatment of TNBC.

## 4. Tumor Metastasis in Triple Negative Breast Cancer

Using CSCs as a prognostic marker in patients with breast cancer has profound clinical significance and has been discussed in one of our prior reviews [17]. The prognostic value of these CSC markers in breast cancer, especially the association with metastasis occurrence and survivals, has been summarized in Table 1. Based on the accumulating evidence, it appears as if combining the three stem cell markers CD44, CD24, and ALDH1 has tremendous value in predicting prognosis, including risk of metastasis and overall survival.

The epithelial–mesenchymal transition (EMT) has been recently recognized to have the ability to convert epithelial cells to mesenchymal cells and subsequently acquire motile and migratory propensity [49]. This propensity, as expected, plays a key role for CSCs to metastasize to distant tissues or organs regardless of chemotherapy [50]. Several signaling pathways in the TME, including, but not limited to, Wnt, Notch, and Shh, as mentioned earlier, produce transcription factors to regulate tumor growth, invasion, and metastasis. If these tumor cells are present in patient vessels and/or lymphatics, they are called circulating tumor cells (CTCs) and have been hypothesized to be involved in the metastatic process [51,52]. Increasing evidence has demonstrated that CSC markers are conserved without alteration during tumor metastasis, with CTCs serving as intermediate cells from primary tumor cells and the distant metastatic tumor cells [53]. BCSCs have been identified in a CTC population among patient peripheral blood samples [54]. Accordingly, this finding makes it possible to detect CTCs by using stem cell markers mentioned above [55,56]. These markers, while indicating the stemness of the CTCs, may be used for the early diagnosis of tumor metastasis, prognosis prediction and therapeutic effect monitoring.

It has been shown that TNBC exhibits more traits possessed by CSC than other breast cancer subtypes and is more likely to develop metastases [57,58]. Driven by the aggregation of CD44+ CSC, more CTC clusters and polyclonal metastasis of TNBC were found to be associated with an unfavorable prognosis [59]. The epidermal growth factor receptor (EGFR) family has been found to be involved in the regulation of cancer metastasis, including TNBC [57,60]. Interactions between the receptor tyrosine kinases EGFR and metastasis with extracellular matrix (ECM)-binding integrins enhance metastatic colonization in model systems [60,61,62]. The current evidence supports dynamic crosstalk between CSCs and metastatic site TME, with contributions by surrounding non-stem cells, including stromal cells, immune cells, and ECM, which are crucial for tumor growth after CTCs seed in the metastatic site [63,64]. CSCs in TNBC have been shown to have characteristic their high invasiveness and metastatic behavior, with increased expression of pro-invasive genes, including those for interleukin (IL)-1, IL-6, IL-8, and urokinase plasminogen activator [64,65].

All the above mechanisms have been shown to be possibly involved in the high propensity for invasiveness and metastasis of BCSCs in TNBC. Targeting these mechanisms can potentially prevent metastasis and therefore improve survival in TNBC.

## 5. Chemoresistance in Triple Negative Breast Cancer

TNBC, the most aggressive and deadliest type of breast cancer, lacks a targeted therapy and has therapy-resistance to the majority of chemotherapy regimens. Since none of the current chemotherapies specifically targets CSCs, CSCs serve as a tumor reservoir for self-renewal and proliferation. This causes inevitable tumor invasion and metastasis, which is more common in TNBC than non-TNBC, ultimately resulting in poor prognosis. Studies have shown CSCs are associated with chemoresistance, particularly in TNBC due to the higher propensity of developing stemness compared to those found in non-TNBC [20,76].

Doxorubicin (Dox), for instance, is widely used in the treatment of TNBC. However, resistance by tumor cells limits its effectiveness. CSCs have been found to be associated with Dox resistance. Signal transducer and activator of transcription 3 (Stat3) and its downstream pathway can convert non-CSCs to CSCs [77]. More recently, a novel signaling pathway that involves Stat3, Oct-4 and c-Myc has been demonstrated to regulate stemness-mediated Dox resistance in TNBC [16,78]. To overcome the mechanism of Dox resistance, WP1066, a Stat3 inhibitor that was more widely studied to treat CNS tumors, is being explored to reduce proliferation of BCSCs in Dox-resistant TNBC [79,80].

Since the CD44+/CD24−/ALDH1+ phenotypes have been recognized as BCSC markers in many studies, a hypothetical approach to targeting CSCs against cell surface membrane antigens such as CD44 would be justified [81,82]. Target options include monoclonal, competitive protein/peptide, HD-CD44 crosstalk, and others [81]. In addition, ALDH1 can be a possible therapeutic target [83], although only limited in vitro data is available through ALDH1 knockdown [84].

Immune evasion has also been suggested to result in chemoresistance in TNBC, as well as tumorigenesis in other tumor types. Tumor-infiltrating lymphocytes (TILs) have been shown to be present in some TNBC and non-TNBC breast cancers and have been associated with favorable prognosis [85,86]. Programmed cell death-1 (PD-1) receptor expression on tumor cells and programmed death-ligand 1 (PD-L1) expression on TILs are therefore indicators of the enrichment of the adaptive immune response against tumor cells. Lack of TIL or PD-1/PD-L1 expression in the tumor microenvironment has been suggested to be associated with less favorable prognosis, especially in early-stage breast cancer [87]. The blockade of PD-1/PD-L1 with checkpoint inhibitors has become a promising immunotherapy to enhance anti-tumor immunity in TNBC after success in treating other types of cancer and is being widely investigated in many ongoing studies.

The involvement of any of the above mechanisms may result in chemoresistance in TNBC. It is essential to take these mechanisms into account when developing novel targeted therapies to overcome chemoresistance.

## 6. Potential Mechanisms/Pathways of Eradicating Breast Cancer Stem Cells and Development of Targeted Therapies

Recent studies have demonstrated the role of BCSCs as the source of metastatic disease and drug resistance in TNBC. Understanding the mechanisms and pathways that stimulate cancer cell proliferation can lead to the development of effective targeted treatment strategies. More importantly, in TNBC, as treatment failure is likely to be associated with BCSCs that possess unique survival mechanisms and pathways that bypass or evade the conventional chemotherapy, developing cancer stem cell-targeted therapies should focus on the specific signaling pathways unique to BCSCs. The three signaling pathways reviewed above, namely the Wnt, Notch, and Shh pathways are potential treatment targets [83]. More and more drugs have been investigated and developed that directly target the Wnt, Notch, and Shh pathways [88]. Treatment with a small molecule β-catenin/TCF inhibitor inhibits β-catenin-mediated transcription, resulting in a reduction in stem cell proliferation and tumor bulking in TNBC cell lines and animal models [89,90]. A selective inhibitor of Wnt, HDAC, and ESR1 has been also shown to be able to suppress the stemness of BSCSs and therefore to convert BCSCs to non-BCSCs in TNBC cells while normal cells in the breast are not affected [38]. Blocking antibodies against the Notch pathway can be categorized into two groups, i.e., those directed to the negative regulatory region (NRR) that enable γ-secretase mediated-cleavage and those that block receptor-ligand interactions by hindering EGF repeats [91,92]. Shh-targeted therapies mostly consist of inhibitors directed against one of the pathway targets, SMO. Although the clinical benefit from SMO inhibitors such as Vismodegib has been established in basal cell carcinoma and medulloblastoma, its clinical effectiveness or benefits towards other solid-type tumors have been limited [93]. GLI transcription factors are the terminal effectors of the Shh-SMO signaling pathway, as discussed above, and are more likely to target CSCs by GANTS (a GLI inhibitor), which is under investigation and has shown some promising in vitro results [88,94]. A summary of the potential pathways specifically targeting BCSCs (CSC-specific mechanisms) is illustrated in Figure 1A.

Non-CSC specific molecular signaling pathways have been proposed to be able to potentially eradicate CSCs and other cancer cells in TNBC. Due to the proven association between the EGFR pathway and TNBC metastasis [95], EGFR inhibitors, such as cetuximab, have been under investigation as a potential adjunct treatment option for metastatic TNBC [96,97]. Notably, although EGFR is relatively non-specific for CSCs, one of its downstream regulators, Stat3, as mentioned earlier, is involved in regulating the self-renewal and stemness of cells, and therefore can be a promising candidate of BCSC-targeted therapy [18,98]. PI3K/Akt/mTOR is another classical pathway that has been found to be associated with many types of cancer. The mechanisms for gene or pathway activation include loss of tumor suppressor gene PTEN function, amplification or mutation of PI3K and/or Akt, and the activation of other inducers such as growth factor receptors and carcinogens [99]. Therapy targeting the PI3K/Akt/mTOR pathway has been suggested to overcome the drug resistance in TNBC by regulating apoptosis in BCSCs in addition to cancer cells [100]. Metformin, a conventional medication for diabetic treatment, has been shown to be able to block the mTOR pathway by activating AMPK adenosinemonophosphate-activated protein kinase (AMPK) [101,102]. Interestingly, it has also been shown to particularly target the CSC population in breast cancer cell lines [103,104,105].

There are a few other mechanisms that have been proposed to tackle therapy-resistant TNBC. These mainly include the blockade or reversal of the EMT of cancer cells and the disruption of TME or the metabolism [106,107,108,109]. Tumors enriched in the carcinoma-associated fibroblasts involved in TME can secrete hepatocyte growth factor (HGF, ligand for c-Met receptor), and C-X-C motif chemokine 12 (CXCL12, THE ligand for chemokine receptor CXCR4) [110]. These factors may contribute to chemoresistance [111]. Dual HDAC and 3-hydroxy-3-methylglutaryl coenzyme A reductase inhibitor, JMF3086, and several other compound drugs have been investigated to alter the EMT of cancer cells and subsequently reduce their plasticity [110]. While therapy leveraging these tumorigenesis mechanisms such as Entinostat may show effectiveness against TNBC [112], it is noted that they are not CSC-specific.

When being treated with conventional chemotherapy, therapy resistant BCSCs, non-BCSC tumor cells, stromal cells and immune cells can result in the minimal residual disease [113,114]. Unlike non-BCSC tumor cells, the stemness of BCSCs allows them to differentiate into multilineages and therefore present with different heterogeneous components within the tumor. The relapsed component, however, can evolve to be more aggressive, evading human immune defense mechanism and the conventional chemotherapy mechanism of action. This acquired characteristics result in early metastasis, particularly in TNBC. To develop targeted therapies for TNBC, a focus-target shifting from non-BCSCs towards BCSCs (the majority of tumor cells) may help overcome multi-drug resistance [111]. In principle, the tumor biological characteristics, BCSC signaling pathways, drug-resistance mechanisms, and the TMEs surrounding the BCSCs are the key factors that can be modified to become a potential treatment options. Several new targets against BCSCs have been identified as promising. Oncotargets such as the cyclin family (D1 and D3) are critical for BCSCs to maintain their clonogenicity. BCSCs have been also shown to be dependent on survival induction factors such as survivin and Myeloid Cell Leukemia 1(MCL1) to extend their survival advantages. Both of these two factors play a role in chemoresistance [73,115]. All these targets have an mRNA sequence that can be modified by mRNA helicase eIF4A [116]. The same authors have shown that eIF4A could downregulate CSC stemness and the levels of ATP-binding cassette transporters that export xenobiotics were significantly reduced [115]. Therefore, eFT226, which targets eIF4A, has become a promising new treatment target option in BCSCs. It may overcome drug resistance by downregulating those drug transporters [111].

Although BCSCs can evade the immune system, resulting in insufficient adaptive and inmate immune responses and subsequent chemoresistance, they are strongly antigenic so that naïve CD8+ effector T-cells, once activated, can still eliminate BCSCs. There is more and more evidence suggesting that empowering the CD8+ effector T-cells through the engagement of their programmed death receptor-1 (PD-1) with the PD1 ligand (PD-L1) would synergize with BCSC-directed therapy and in a combination with other target therapies [117,118]. A summary of the non-CSC specific mechanisms and pathways targeting breast cancer cell is illustrated in Figure 1B.

Lastly, gene therapy using advanced gene editing technology such as CRISPR targeting CSCs in TNBC could be a potential treatment option. The CRISPR/Cas9 system has been emerged as a leading technology to more quickly target specific genetic loci and more accurately modify genes of interest [119]. Castro et al. developed a novel TNBC model in mice and discovered Cripto-1 as a target for TNBC tumor cells that can be genetically modified by the CRISPR/Cas9 system [21]. Hypothetically, the system can target any genes that involve cancer cell stemness and therefore serves as a versatile treatment strategy for TNBC.

Table 2 summarizes the current clinical trials in chemoresistant breast cancer excluding PD-1/PD-L1 trials since they have been extensively reviewed in the literature [120,121,122,123]. Also note that poly (ADP-ribose) polymerase (PARP) and cyclin-dependent kinase (CDK) 4/6 play a key role in cell cycle, and are neither specific to breast cancer cells or CSCs. Although several trials found on clinicaltrials.gov use PARP inhibitors and CDK4/6 inhibitors, their mechanism is less relevant to the theme of this review and is therefore not discussed.

## 7. Conclusions

TNBC remains an aggressive disease due to the lack of targeted treatment and low rate of response to chemotherapy. BCSCs are a small subpopulation of breast tumors that play a vital role in metastatic disease and drug resistance. TNBC tumors are characterized as being enriched for BCSCs.

Recent advances in BCSC biology in TNBC and the identification of cancer stem cell biomarkers have paved the way for the development of cancer stem cell-targeted therapies.

Multiple targets against BCSCs are currently under investigation with the goals of either selectively targeting BCSCs or co-targeting BCSCs and non-BCSCs (majority of tumor cells).

We hope this review provides insights into mutational landscape of BCSCs and the discovery of potential molecular signaling pathways targeting BCSCs to overcome chemoresistance and prevent metastasis, ultimately to improve the prognosis of TNBC.

## 8. Future Direction

With the growing evidence of the role of BCSCs in TNBC, it is important to better define their biological characteristics, molecular pathways that sustain stemness, and mechanisms of drug resistance to discover effective cancer stem cell targeted therapies.

The identification and quantification of existence of BCSCs using stem cell markers and deployment of CSC-targeted therapies may revolutionize the TNBC treatment paradigm. BCSCs may become potential prognostic and predictive biomarkers for metastasis and chemoresistance, and eradicating BCSCs in the TNBC may improve the overall survival of these patients. Although there have been more promising results recently, the shortcomings of the current research on BCSCs include a limited number of biomarkers and therapeutic targets. More clinical trials are also needed to demonstrate effectiveness and safety profiles of the new investigational drugs.

## Figures and Tables

**Table 1:** Summary of studies on metastasis occurrence and survivals in stem like breast cancer.

Study	TNBC	All Cases	Metastasis Occurrence	Survival
(Abraham et al., 2005) [66]	NA	112	12 (80%) cases with CD44+/CD24−/low phenotype had distant metastasis (*p* = 0.04). All 5 cases with more than 20% CD44+/CD24−/low tumor cells had osseous metastasis (*p* = 0.02).	The percentage of CD44+/CD24−/low tumor cells had no influence on DFS or OS
(Liu et al., 2007) [10]	N/A	581	The IGS * in CD44+/CD24−/low cancer cells was significantly associated with the risk of metastasis regardless of tumor size or lymph-node status (*p* < 0.05).	Of patients treated with chemotherapy, IGS in CD44+/CD24−/low cancer cells was associated with lower 10-year metastasis-free survival (*p* < 0.001).
(Lin et al., 2012) [67]	62	147	The proportion of CD44+/CD24− tumor cells was correlated with lymph node involvement (*p* = 0.026).	The proportion of CD44+/CD24− tumor cells was significantly associated with DFS (*p* = 0.002) and OS (*p* = 0.001).
(Adamczyk et al., 2014) [68]	35	156	NA	In patients treated with anthracyclines and taxanes, significantly longer survival was associated with CD44+ phenotype (DFS *p* = 0.019, OS *p* = 0.062) and CD44+/CD24− phenotype (DFS *p*=0.006, OS *p* = 0.019).
(Chen et al., 2015) [69]	21	140	The proportion of CD44+/CD24− tumor cells was significantly associated with lymph node involvement (*p* = 0.016), distant metastasis (*p* = 0.001), and recurrence (*p* = 0.013)	High CD44+/CD24− phenotype had worse response to chemotherapy (*p* = 0.001), and worse DFS (*p* = 0.0012) and OS (*p* = 0.017)
(Collina et al., 2015) [70]	160	160	Only CD44, not CD24, CD133, ALDH1 and ABCG2, was significantly associated with metastases (*p* = 0.011).	Among CD44, CD24, CD133, ALDH1 and ABCG2, only CD44 was significantly associated with DFS (*p* = 0.051).
(Wang et al., 2017) [71]	67	67	CD44+/CD24− subtype possessed slightly increased risk of metastasis or recurrence compared with CD44−/CD24− subtype.	CD44+/CD24− tumor cells were associated with worse OS (*p* = 0.005).
(Ma et al., 2017) [72]	158	158	ALDH1 expression was significantly correlated with tumor stage (*p* = 0.04).	ALDH1 expression was associated with shorter RFS (*p* = 0.01) and OS (*p* = 0.001).
(Lee & Kim, 2018) [73]	1	2	Both cutaneous metastatic cases had high expression of CD44+/CD24− and ALDH1+.	NA
(Rabinovich et al., 2018) [74]	31	144	NA	CD44+/CD24− phenotype was associated with a greater risk of relapse (*p* = 0.011) and a worse outcome (*p* = 0.019). TNBC was associated with ALDH+ (*p* = 0.039).
(Althobiti et al., 2020) [75]	178	930	NA	The high expression of ALDH1 was significantly associated with poor survival (*p* < 0.001), and particularly in the luminal B (*p* = 0.042) and TNBC (*p* = 0.003) subtypes.

* IGS: invasiveness gene signature; DFS: disease-free survival; OS: overall survival; RFS: relapse-free survival; NA: not available.

**Table 2:** Clinical trials treating chemoresistant breast cancer registered on clinicaltrials.gov.

NCT Number	Conditions	Drug of Interest	Drug Category	Phases	Enrollment	Study Designs	Country
NCT02158507	Metastatic TNBC	Veliparib	PARP inhibitor		23	Single group	USA
NCT04134884	Metastatic Breast Cancer	Talazoparib	PARP inhibitor	1	38	Sequential	USA
NCT01477060	Metastatic Breast Cancer	Metformin	AMPK agonist	2	32	RCT ^#^	Italy
NCT02299635	TNBC	PF-03084014	γ-secretase inhibitor	2	19		Multiple
NCT03361800	TNBC	Entinostat	HDAC inhibitor	1	5	Single group	USA
NCT04333706	TNBC	Sarilumab	IL-6R	1/2	65	Non-Randomized; Parallel	USA
NCT04360941	Advanced Breast Cancer	Palbociclib	CDK4/6 inhibitor	1	45	Single group	UK
NCT03218826	Advanced Breast Cancer	AZD8186	PI3K inhibitor	1	58	Single group	USA
NCT03853707	Advanced Breast Cancer	Ipatasertib	Akt inhibitor	1/2	40	RCT	USA
NCT03979508	Breast Cancer	Abemaciclib	CDK4/6 inhibitor	2	100	Non-Randomized; Parallel	USA
NCT03740893	Breast Cancer	AZD6738/Olaparib	ATR * kinase inhibitor/PARP inhibitor	2	81	RCT	UK
NCT01617668	Breast Cancer	LCL161	SMAC ^^^ mimetic	2	209	RCT	Multiple
NCT01266486	Breast Cancer	Metformin	AMPK agonist	2	41	Single group	UK
NCT04092673	Solid Tumor (w/Breast Cancer)	eFT226	eIF4A Inhibitor	1/2	45	Sequential	USA

* ATR: ataxia telangiectasia and Rad3 related; ^^^ second mitochondria-derived activator of caspases; ^#^ RCT: randomized controlled trial.
